# The prevalence and infection rates of amphistome species in intermediate snail hosts: a systematic review and meta-analysis

**DOI:** 10.3389/fvets.2024.1418979

**Published:** 2024-06-17

**Authors:** Ignore Nyagura, Mokgadi Pulane Malatji, Samson Mukaratirwa

**Affiliations:** ^1^School of Life Sciences, College of Agriculture, Engineering and Science, University of KwaZulu-Natal, Westville Campus, Durban, South Africa; ^2^One Health Centre for Zoonoses and Tropical Veterinary Medicine, Ross University School of Veterinary Medicine, Basseterre, Saint Kitts and Nevis

**Keywords:** amphistomes, intermediate host snails, meta-analysis, prevalence, natural infection, experimental infection

## Abstract

The systematic review and meta-analysis were conducted to determine the estimates of the prevalence and infection rates of natural and experimental infections of amphistome species in intermediate host snails (IHs) across different continents. A search of peer-reviewed literature on natural and experimental infections of freshwater snails with amphistome species was conducted from four electronic databases from 1984 to 2023. The estimates of the prevalence and/or infection rates were based on 36 eligible peer-reviewed articles, which met the inclusion criteria and reported on natural and experimental infections of amphistome species in freshwater snails. The results showed that a total of 1,67,081 snail species from the peer-reviewed articles were examined for natural infections and 7,659 snail species for experimental infections. The overall pooled prevalence of amphistome infections from naturally infected snails was 2% (95% CI: 0–4), while the overall pooled prevalence of amphistome infections from infections was 40% (95% CI: 18–64). The highest pooled prevalence of natural infection was 3%, which was recorded in Europe (95% CI: 1–7%). The highest overall prevalence of naturally infected amphistome was 6% (95% CI: 0–20%) for *Paramphistomum epiclitum*. The Americas had the highest pooled prevalence of experimental amphistome infection among freshwater snails (66%; 95% CI: 26–96%). The highest pooled infection rate of 65% (95% CI: 12–100%) was recorded for *Paramphistomum cervi* in experimental infections. *Galba truncatula* was the only snail that qualified for meta-analysis for natural infection with *Calicophoron daubneyi,* with a pooled prevalence of 3% (95% CI: 1–8%). *Galba truncatula* infected with *C. daubneyi and P. cervi,* and *Bulinus tropicus* infected with *Calicophoron microbothrium* in the experimental infection qualified for the meta-analysis, with an overall infection rate of 66% (95% CI: 34–92%) and 30% (95% CI: 0–74%), respectively. The pooled prevalence of amphistome species infection in the intermediate host (IH) snails based on detection techniques was higher with PCR compared to the dissection and shedding of cercariae. The results from the quality effects model revealed a high heterogeneity and publication bias between studies. This meta-analysis provided valuable insights into the prevalence and infection rates of amphistome species in snail IHs across different geographical regions.

## Introduction

Intestinal amphistomosis, otherwise known as amphistomiasis (1), paramphistomiasis (2, 3), or paramphistomosis (4), is a neglected trematode disease of domestic and wild ruminants (1, 5, 6). The disease is caused by a heavy infection with immature flukes, resulting in acute gastroenteritis with significant morbidity and mortality, particularly in young animals (1). In domestic ruminants, amphistomosis can cause major economic losses due to decreased milk and meat production, poor nutrient conversion, weight loss, and reduced fertility (2).

The disease is spread worldwide, with the highest infection rates reported in the tropical and subtropical regions of Asia (2, 7), Africa (2, 6, 8), Australia (2), Europe (2, 4), and Russia (2). The causative amphistome species are commonly known as rumen flukes or conical flukes, belonging to the class Trematoda under the superfamily Paramphistomoidea Fischoeder, 1901 (9, 10). This superfamily is composed of 100 s of species belonging to 10 families: Balanorchiidae Stunkard, 1925; Cladorchiidae Fischoeder, 1901; Diplodiscidae Cohn, 1904; Gastrothylacidae Stiles and Goldberger, 1910; Gastrodiscidae Monticelli, 1892; Olveriidae Yamaguti, 1958; Paramphistomidae Fischoeder, 1901; Stephanopharyngidae Stiles and Goldberger, 1910; Zonocotylidae Yamaguti, 1963; and Zygocotylidae Yamaguti, 1963. The group of trematodes is characterized by the absence of an oral sucker and the position of the acetabulum at or close to the posterior end of the body in both adults and cercariae (10).

Amphistomes have a complex heteroxenous life cycle that involves a variety of species of intermediate host snails (IHs), where development and asexual replication occur, giving rise to multiple cercariae that escape the snail and encyst on the water surface, plants immersed in water or herbage to form metacercariae. Domesticated and wild ruminants are definitive hosts that become infected through drinking water or grazing on pasture contaminated with metacercariae (1, 2, 11). Within the definitive hosts, the prepatent period ranges between 56 and 89 days (1). Adult flukes located in the rumen and reticulum produce eggs, which are then passed through feces (1, 2). The eggs that need an aquatic environment hatch into miracidia, which is the infective stage for the intermediate hosts, and the miracidia will infect various intermediate host snails (6).

To date, there are more than 70 amphistome species reported in both domestic and wild ruminants worldwide (1, 6, 12). The IHs belonging to the taxa Caenogastropoda Cox, 1960; Stylommatophora Schmidt, 1855; and Basommatophora Keferstein, 1864, involving many genera and species within the families Planorbidae Rafinesque, 1815, and Lymnaeidae Rafinesque, 1815 (13) have been implicated in the transmission of various amphistome species. These snails play a vital role as obligatory intermediate hosts in the transmission of trematodes to their final hosts (3, 14–20).

The prevalence of amphistome infections or any snail-borne trematode is influenced by the abundance of susceptible IHs and vertebrate definitive hosts (1). Thus, the availability of the IHs in the grazing habits of the infected vertebrate definitive hosts largely determines the epidemiology and seasonal patterns of amphistome infection (2, 21). Furthermore, knowledge of the IHs for various amphistome species is essential in developing sustainable strategies for the prevention and control of amphistome infections in domestic and wild ruminants, especially in areas where mixed livestock and game farming are being practiced. Therefore, this study systematically reviewed and analyzed quantitative data extracted from peer-reviewed articles on the prevalence and infection rates of natural and experimental infections of amphistome species in various snail host species from 1984 to 2023.

## Methods

### Search strategy

A systematic literature search was conducted on Google Scholar, PubMed, Scopus, and Web of Science Core Collection databases to retrieve studies from 1984 to 2023 using Boolean Operators (AND, OR) and a combination of keywords such as amphistome OR paramphistome AND intermediate host snail OR freshwater snail OR gastropods AND natural infections AND prevalence, amphistome OR paramphistome AND intermediate host snail OR freshwater snail OR gastropods AND experimental infections OR infectivity OR susceptibility AND prevalence. Additional relevant studies were identified by cross-referencing or screening through bibliographies (snowballing) of selected articles. EndNote reference management version X8 (Clarivate Analytics, Philadelphia, PA, USA) was used to retrieve and manage full-text articles.

### Inclusion and exclusion criteria

Five inclusion criteria were used to select articles for the systematic review and meta-analysis from 1984 to 2023, which are listed as follows: (i) studies reporting the number of screened and infected snails with amphistome species, (ii) studies reporting intermediate host snails to species level, (iii) studies reporting data on amphistome infections to species level, (iv) studies that reported prevalence and/or infection rates based on natural and/or experimental infections, and (v) studies that mentioned the detection method(s) of amphistome infection in snails.

All studies reporting on other trematode species including amphistome infections, but did not identify amphistomes and/or the intermediate hosts up to species level or reporting on amphistome infections in definitive hosts were excluded. Furthermore, articles published in other languages besides English or outside the period of study were excluded.

### Data extraction

Following the study design, the title and abstract of the articles were assessed independently by two reviewers (IN and MPM), and relevant articles were retrieved. The retrieved articles were thoroughly rechecked, and duplicates and articles that did not fulfill the inclusion criteria were removed. For meta-analysis, data were extracted from text, tables, and figures and processed in MS Excel. Variables that were considered for each article are as follows: author names, year of publication, year of study and duration, country of study, snail species, amphistome species, detection method(s), number of snail samples screened, number of positives, and prevalence rate.

### Quality assessment of the articles

The Grading of Recommendations Assessment, Development, and Evaluation (GRADE) approach was used to assess the overall quality of articles for meta-analysis (22, 23). Articles that met each given inclusion criteria received one point, and as a result, each study was assigned a score ranging from 0 to 5. Publications with a total score of 5 points were deemed as good quality, 4 as moderate quality, and ≤ 3 as low quality and were excluded (23). The standardized quality index score (ranging from 0 to 1) was then computed (Table 1).

**Table 1:** Frequency of snail species naturally infected with amphistomes from 1984 to 2023.

Snail species	No. of studies	No examined	No infected	Prevalence (%)
*Biomphalaria (Bio.) peregrina*	1	1,424	8	0.56
*Bio. tenagophila*	1	4,448	10	0.22
*Bithynia (Bi.) tentaculata*	2	3,180	15	0.47
*Bulinus (B.) tropicus*	2	406	84	20.69
*Ferrissia (F.) fragilis*	1	243	5	2.06
*Galba (G.) truncatula*	13	95,839	4,644	4.85
*Gyraulus (Gy.) convexiusculus*	2	6,677	169	2.53
*Helicorbis (H.) coenosus*	1	474	0	0.00
*Helisoma (He.) anceps*	1	233	1	0.43
*Indoplanorbis (I.) exustus*	2	4,232	434	10.26
*Lymnaea (L.) acuminata*	1	699	0	0.00
*L. luteola*	2	3,064	56	1.83
*L. ovata*	1	108	5	4.63
*Melanoides (M.) tuberculata*	1	32,026	8	0.02
*Menetus (Me.) dilatatus*	1	494	4	0.81
*Omphiscola (O.) glabra*	3	9,963	270	2.71
*Physa (Ph.) acuta*	2	430	7	1.63
*Planorbis (P.) leucostoma*	1	1841	0	0.00
*P. planorbis*	1	50	0	0.00
*Pseudosuccinea (Ps.) columella*	1	971	1	0.10
*Vivipara (V.) bengalensis*	1	279	0	0.00
Total		167,081	5,721	3.42

### Study quality assessment, data preparation, and analysis

Prevalence data were transformed using the double arcsine method to avoid overestimating the weight of individual studies (24). The double arcsine approach uses the arcsine transformation twice to measure the prevalence of the meta-analysis and to take into account the heterogeneity caused by studies with small sample sizes and extreme proportions (24). The MetaXL add-in for Microsoft Excel^1^ was used to compute a quality effects model to account for the heterogeneity. The heterogeneity between estimates was quantified using the inverse variance statistic (*I^2^* index), and its significance was assessed using the Cochrane’s Q test. Following the protocol of Higgins et al. (25), the *I^2^* score of 25, 50%, or 75% was interpreted as low, moderate, or high heterogeneity, respectively. Forest plots were used to graphically demonstrate the estimated prevalence and the 95% confidence interval of the amphistome species among the intermediate host snails. A subgroup analysis was conducted to evaluate the estimates of the prevalence for the major subgroups, including geography (continent), snail species, amphistome species, detection method, and years. Funnel plots were used to evaluate the publication bias.

## Results

### Search results

**Figure 1:**
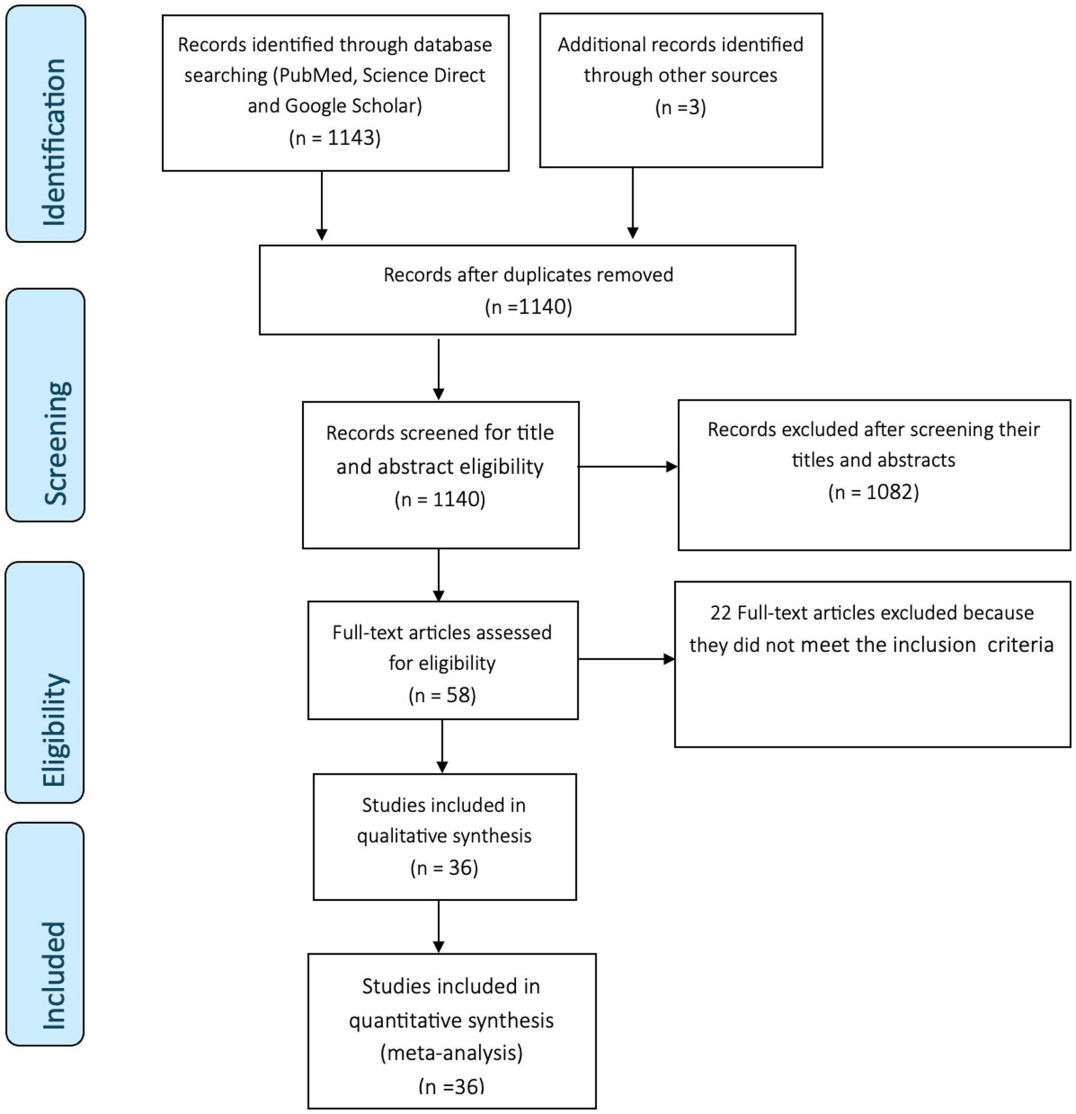
PRISMA flow diagram.

A total of 1,146 records, from which 36 were found to be eligible articles, were identified through database searching and snowballing, as summarized in Figure 1. Preliminary screening of duplicates, titles, and abstracts deemed 1,088 articles irrelevant and were excluded. Full-texts of 58 articles were assessed for eligibility, and 22 articles were excluded as they did not meet the inclusion criteria. The remaining 36 articles distributed across 5 continents met the inclusion criteria and quality assessment. In total, 20 articles (55.6%) were field-based studies, and 16 (44.4%) were experimental studies. Of the field studies conducted, twelve (60%) were from Europe, four (20%) were from Asia, two (10%) were from Africa, and one (5%) each from North America and South America. The geographical distribution and contributions of the experimental studies were as follows: Europe (43.8%), Africa (25%), North America (12.5%), South America (12.5%), and a combination of Africa and Europe (6.3%). The total quality of all primary studies was five, indicating that the quality of the included studies was high.

### Natural infections of amphistomes in freshwater snails

The prevalence of amphistome cercaria in field-collected snails from the studies ranged from 0 to 22.54% (Supplementary Table S1). A total of 167,081 freshwater snails represented by 21 different snail species were collected and identified (Table 1). Out of these snails, 5,721 were infected with different amphistome species. The results from the natural infection showed that 16 species of IHs were implicated in the transmission of 10 amphistome species (*Calicophoron [C.] daubneyi, C. microbothrium, Explanatum [E.] explanatum, Fischoederius [F.] elongatus, Gastrothylax [G.] crumenifer, Orthocoelium [O.] scoliocoelium, Paramphistomum [P.] epiclitum, Pisciamphistoma [Pi.] stunkardi, Stichorchis [S.] subtriquetrus,* and *Zygocotyle [Z.] lunata*) (Table 2).

**Table 2:** Documented snail species naturally infected with various amphistome species from 1984 to 2023.

Snail species	*N*	Number of snails naturally infected with various amphistome species									
		*Z. lunata*	*G. crumenifer*	*O. scoliocoelium*	*C. daubneyi*	*C. microbothrium*	*S. subtriquetrus*	*E. explanatum*	*F. elongatus*	*P. epiclitum*	*Pi. stunkardi*
*Bio. peregrina*	1,424	8	–	–	–	–	–	–	–	–	–
*Bio. tenagophila*	4,448	10	–	–	–	–	–	–	–	–	–
*Bi. tentaculata*	3,180	–	5	10	–	–	–	–	–	–	–
*B. tropicus*	406	–	–	–	–	84	–	–	–	–	–
*F. fragilis*	243	–	–	–	–	–	5	–	–	–	-
*G. truncatula*	95,839	–	–	–	4,644	–	–	–	–	–	–
*Gy. convexiusculus*	6,677	–	110	–	–	–	–	1,613	––	–	–
*H. coenosus*	474	–	0	0	–	–	–	0	0	0	–
*He. anceps*	233	–	–	–	–	–	1	–	–	–	–
*I. exustus*	4,232	–	–	–	–	–	–	–	–	434	–
*L. acuminata*	699	–	0	0	–	–		0	0	0	–
*L. luteola*	3,064	–	–	–	–	–	–	–	56	–	–
*L. ovata*	108	–	–	–	5	–	–	–	–	–	–
*M. tuberculata*	32,026	–	8	–	–	–	–	–	–	–	–
*Men. dilatatus*	494	–	–	–	–	–	–	–	–	–	4
*O. glabra*	13,863	–	–	–	270	––	–	–	–	–	–
*Ph. acuta*	430	–	–	–	7	–	–	–	–	–	–
*P. leucostoma*	1841	–	–	–	0	–	–	–	–	–	–
*P. planorbis*	50	–	–	–	–	–	–	–	–	–	–
*Ps. columella*	971	–	–	–	–	–	1	–	–	–	–
*V. bengalensis*	279	–	0	0	–	–	–	0	0	0	–

#### The overall prevalence of natural infections of amphistome species in freshwater snails

The results from the quality effects model revealed high heterogeneity (*Q* = 9639.15, *p* < 0.001), and the *I*^2^ index was 99% (Supplementary Figure S1). The overall pooled prevalence of amphistome infection from naturally infected snails was 2% (95% CI: 0–4) (Supplementary Figure S1), and *C. daubneyi* was the most frequently reported infection in *Galba (G.) truncatula* and was found predominantly in Europe.

#### The prevalence of natural infection of amphistomes in freshwater snails by continent

**Figure 2:**
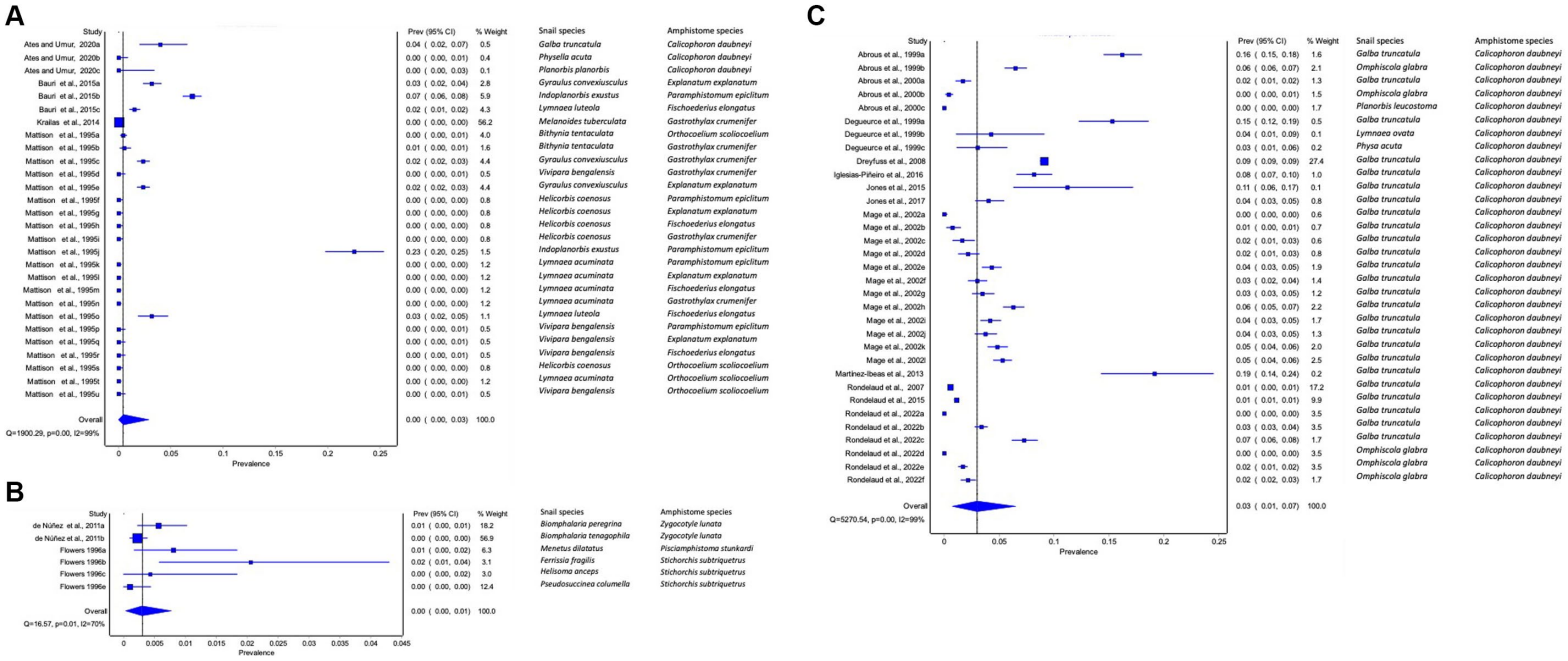
Forest plots of the prevalence of amphistome species in intermediate host snails from natural infection from **(A)** Asia, **(B)** America, and **(C)** Europe recorded from 1984 to 2023.

The pooled prevalence of amphistome species among freshwater snails varied from continent to continent. The pooled prevalence of amphistome species infection among freshwater snails was low in Asia (95% CI: 0–3%, Figure 2A) and in the Americas (95% CI: 0–1%, Figure 2B) and high in Europe (3, 95% CI: 1–7%, Figure 2C). Data from North and South America were combined as Americas for meta-analysis due to their proximity, and Africa as a continent did not have sufficient data for meta-analysis, as only two studies were published, each demonstrating the infection of *C. microbothrium* in *Bulinus (B.) tropicus* with the prevalence of 16.36 and 21.37%, respectively.

#### The prevalence of natural infection of amphistomes in freshwater snails by amphistome species

The pooled prevalence of individual amphistome species in snail intermediate host species was computed (Table 3), with *C. daubneyi* prevalence estimated at 3% (95% CI: 1–6%). *Calicophoron daubneyi* was the most prevalent amphistome species in snails from Europe and in a single study in Asia. The highest overall prevalence estimated from snails in Asia was for *P. epiclitum* at 6% (95% CI: 0–20%), and the prevalence was substantially low for *O. scoliocoelium* (95% CI: 0–1%), *G. crumenifer* (95% CI: 0–2%), and *F. elongatus* (95% CI: 0–2%). Only *S. subtriquetrus,* with an overall prevalence of 6% (95% CI: 0–20%) in North America, qualified for meta-analysis. Three studies from Africa and one study from Asia reported on the prevalence of *C. microbothrium* from *Bulinus* species, and the estimated prevalence was 5% (95% CI: 0–15%). The results from the quality effects model revealed a high heterogeneity between studies on amphistomes with *I*^2^ > 75 except for *E. explanatum* with *I*^2^ = 19% and *O. scoliocoelium* with *I*^2^ = 60. It was not possible to conduct a meta-analysis for amphistome species, such as *Pi. Stunkardi* and *Z. lunata,* since they were surveyed in a limited number of studies and host species.

**Table 3:** Pooled prevalences (PPs) of amphistome species in snail species from natural infections across different continents (1984–2023).

Amphistome species	Continent	No of studies	No of snail hosts	PP (PP) (95% CI)	*I*^2^ (%)
*E. explanatum*	Asia	2	4	3% (95% CI: 2–3%).	19
*C. daubneyi*	Europe and Asia	13	6	3% (95% CI: 1–6%)	99
*C. microbothrium*	Africa and Asia	4	3	5% (95% CI: 0–15%).	98
*F. elongatus*	Asia	2	5	0% (95% CI: 0–2%).	97
*G. crumenifer*	Asia	2	6	0% (95% CI: 0–2%),	97
*O. scoliocoelium*	Asia	1	4	0% (95% CI: 0–1%),	60
*P. epiclitum*	Asia	2	5	6% (95% CI: 0–20%)	99
*Pi. stunkardi*	North America	1	1	N/A	N/A
*S. subtriquetrus*	North America	1	3	6% (95% CI: 0–20%)	80
*Z. lunata*	South America	1	2	N/A	N/A

#### The prevalence of natural infection of amphistomes in snail species

The frequency of infected snail species with different amphistome species is presented in Table 1. The snail species that qualified for meta-analysis was *G. truncatula* infected with *C. daubneyi* with a pooled prevalence of 3% (95% CI: 1–8%), and a high level of heterogeneity was observed (*I*^2^ = 99%). Other infected snails were investigated for a few amphistome species and only in a few studies; thus, it was not possible to carry out a meta-analysis.

#### The prevalence of natural infection of amphistomes in freshwater snails by time in years

**Figure 3:**
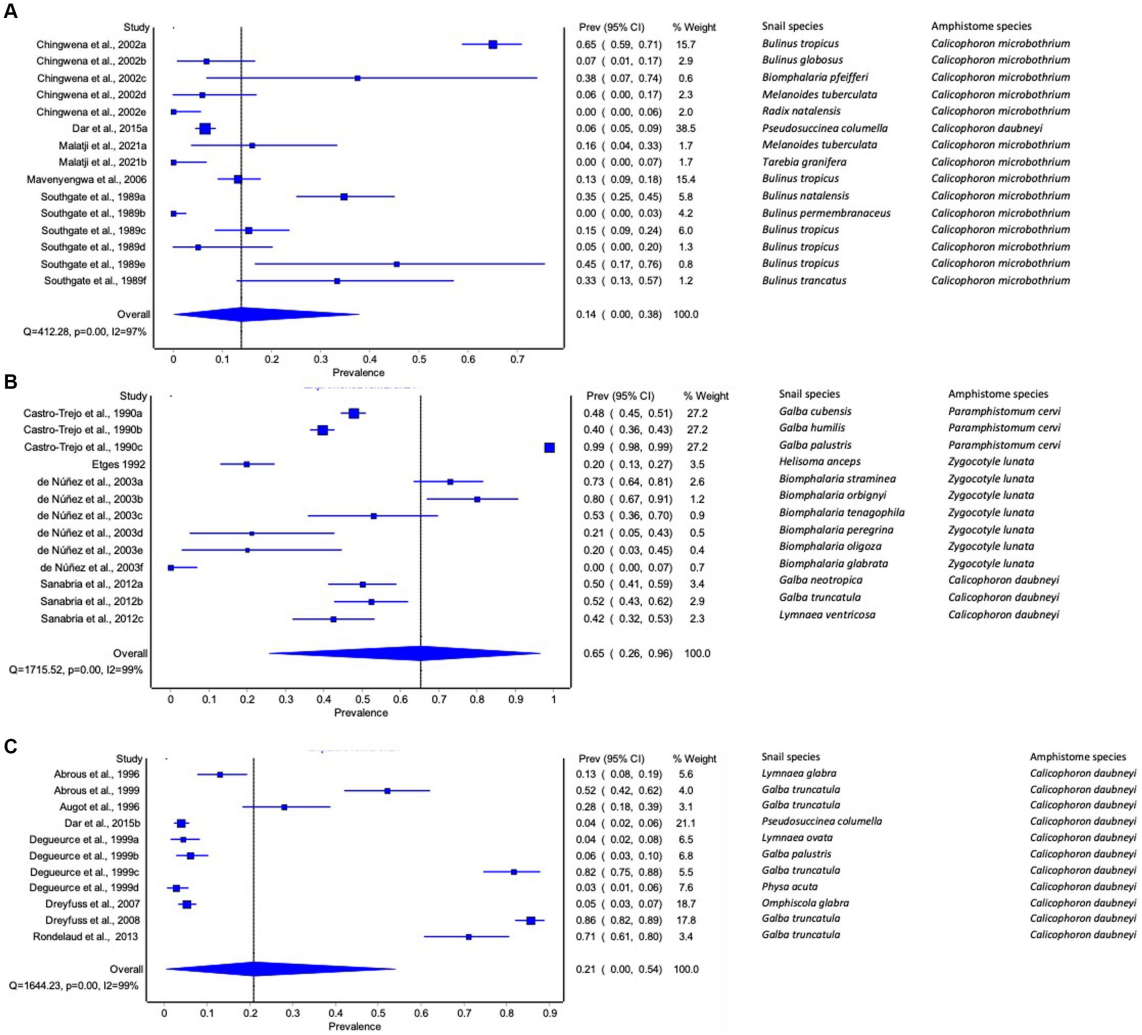
Forest plots of experimental infection rate of amphistome species in intermediate host snails from **(A)** Africa, **(B)** America, and **(C)** Europe recorded from 1984 to 2023.

To evaluate the changes in amphistome infection rates in intermediate host snails between the eras, articles were divided into four groups based on decade: 1984–1993, 1994–2003, 2004–2013, and 2014–2023. Most studies were conducted between 1994 and 2003, and studies between 1984 and 1993 were not eligible for meta-analysis. The pooled prevalence of amphistome species among freshwater snails was higher in years 2004–2013 (4%; 95% CI: 0–14%), followed by 1994–2003 (2%; 95% CI: 1–4%) and 2014–2023 (1%; 95% CI: 0–3%) (Supplementary Figures S2A–C). The results from the quality effects model revealed a high heterogeneity between studies on each given period, 1994 to 2003 (*I*^2^ = 98%), 2004 to 2013 (*I*^2^ = 100%), and 2014 to 2023 (*I*^2^ = 99%) (Figures 3A–C).

#### The prevalence of natural infection of amphistomes in freshwater snails by detection techniques

**Figure 4:**
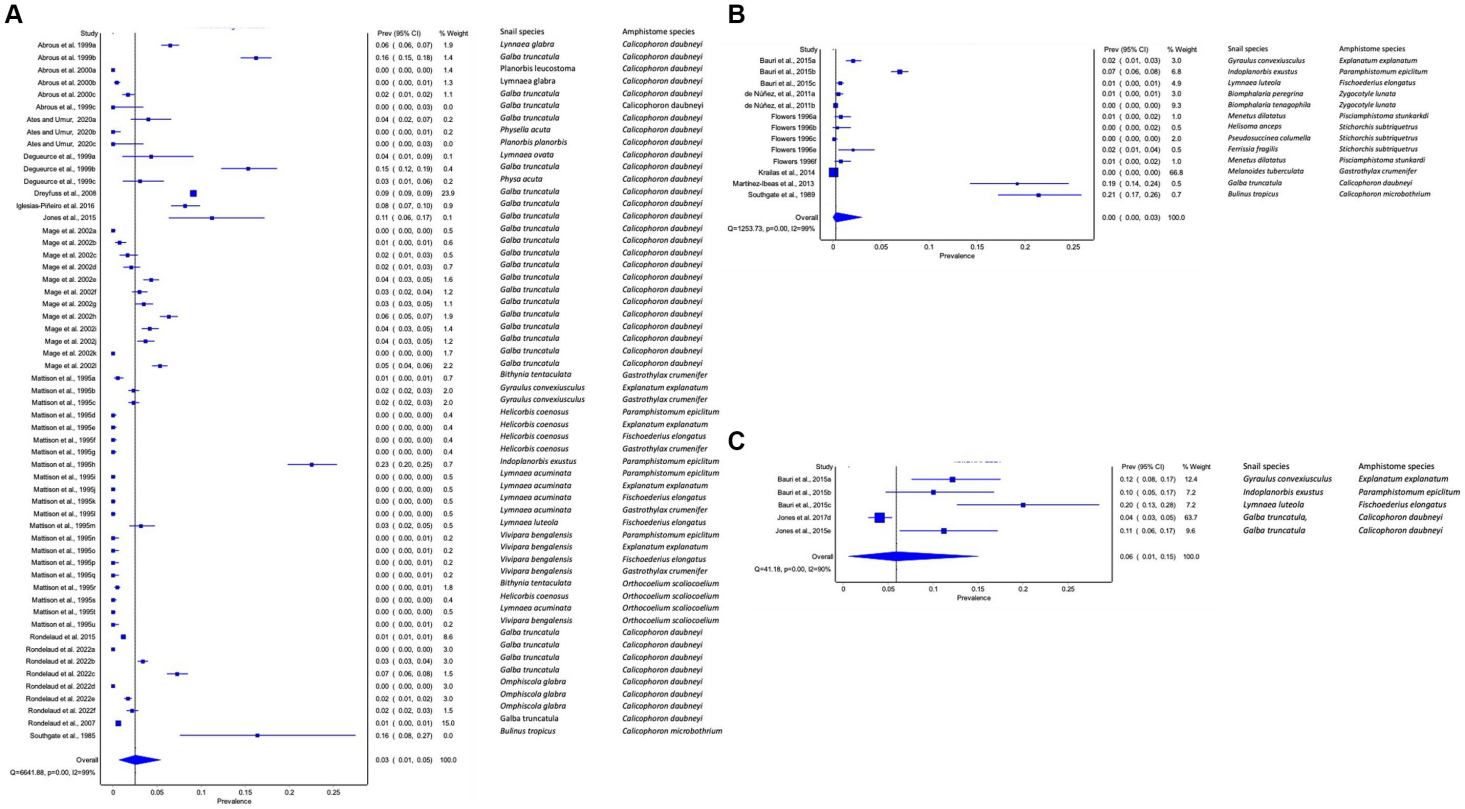
Forest plots of the prevalence of natural infections of amphistome species in intermediate host snails as determined by **(A)** dissection, **(B)** shedding, and **(C)** molecular methods.

To detect natural infections in the intermediate host snails, 12 studies used dissection, 8 studies used cercarial shedding, 2 studies used molecular (PCR) (Figures 4A–C), and one study involved both dissection and molecular. The pooled prevalence of natural infections of amphistomes in the intermediate host (IH) snails based on techniques was higher with PCR (6, 95% CI: 1–15%) (Figure 4C), followed by dissection with 3% (1–5%) (Figure 4A) and cercarial shedding with (95% CI: 0–3%) (Figure 4B). The results revealed a high heterogeneity between studies involving dissection (*I*^2^ = 99%), shedding (*I*^2^ = 99%), and PCR (*I*^2^ = 90%) (Figures 4A–C).

#### Publication bias of studies reporting on natural infections of amphistomes in intermediate host snails

The visual inspection of the funnel plots revealed an asymmetric funnel shape, demonstrating the presence of publication bias (Supplementary Figure S3), which may be due to a small sample size bias or publication bias in the articles.

### Experimental infection rates of amphistomes in freshwater snails

The infection rate of amphistome cercaria in experimentally infected snails from individual studies ranged from 0 to 100% (Supplementary Table S2). A total of 7,659 snails were collected and used in the experiments, and out of these snails, 3,163 were challenged with different amphistome species (Table 4). The 27 snail species included in the experimental studies were *B. glabrata, B. oligoza, B. orbignyi, B. peregrina, B. pfeifferi, B. straminea, B. tenagophila, B. globosus, B. natalensis, B. permembranaceus, B. truncatus, B. tropicus, G. truncatula, G. cubensis, G. humilis, G. neotropica, G. palustris, He. anceps, Lymnaea (L.) glabra, L. ovata, L. ventricose, M. tuberculata, Omphiscola (O.) glabra, Ph. acuta, Ps. columella, Radix (R.) natalensis,* and *Tarebia (T.) granifera* (Table 4). Of these snail species, 23 species were susceptible to infection with a specific amphistome species, including *C. daubneyi,* which caused infections in nine snail species, *C. microbothrium* in six snail species, *P. cervi* in three snail species, and *Z. lunata* in six snail species (Table 5).

**Table 4:** Frequency of snail species experimentally infected with amphistomes from 1984 to 2023.

Snail species	No of studies	No examined	No infected	Infection rate (%)
*Bio. glabrata*	1	25	0	0.00
*Bio. oligoza*	1	15	3	20.00
*Bio, orbignyi*	1	45	36	80.00
*Bio. peregrina*	1	19	4	21.05
*Bio. pfeifferi*	1	8	3	37.50
*Bio. straminea*	1	96	70	72.92
*Bio. tenagophila*	1	34	18	52.94
*B. globosus*	1	44	3	6.82
*B. natalensis*	1	89	31	34.83
*B. permembranaceus*	1	64	0	0.00
*B. trancatus*	1	18	6	33.33
*B. tropicus*	3	597	207	34.67
*G. truncatula*	6	934	671	71.84
*G. cubensis*	1	1,000	477	47.70
*G. humilis*	1	1,000	395	39.50
*G. neotropica*	1	126	63	50.00
*G. palustris*	2	1,166	997	85.51
*He. anceps*	1	127	25	19.69
*L. glabra*	1	138	18	13.04
*L. ovata*	1	159	7	4.40
*L. ventricosa*	1	85	36	42.35
*M. tuberculata*	2	59	6	10.17
*O. glabra*	1	461	24	5.21
*Ph. Acuta*	1	187	5	2.67
*Ps. columella*	1	1,108	58	5.23
*R. natalensis*	1	30	0	0.00
*T. granifera*	1	25	0	0.00
Total		7,659	3,163	41.30

**Table 5:** Documented snail species experimentally infected with various amphistome species from 1984 to 2023.

Snail species	*N*	Number of cases experimentally infected with various amphistome species			
		*C. daubneyi*	*C. microbothrium*	*P. cervi*	*Z. lunata*
*Bio. glabrata*	25	–	–	–	0
*Bio. oligoza*	15	–	–	–	3
*Bio. orbignyi*	45	–	–	–	36
*Bio. peregrina*	19	–	–	–	4
*Bio. pfeifferi*	8	0	3		
*Bio. straminea*	96	–	–	–	70
*Bio. tenagophila*	34	–	–	–	18
*B. globosus*	44	–	3	–	–
*B. natalensis*	89	–	31	–	–
*B. permembranaceus*	64	–	0	–	–
*B. trancatus*	18	–	6		–
*B. tropicus*	597	0	207		–
*G. cubensis*	1,000	–	–	477	–
*G. humilis*	1,000	–	–	395	–
*G. neotropica*	126	63	–		–
*G. palustris*	1,166	10	–	987	–
*G. truncatula*	934	671	–	–	–
*H. anceps*	127	–	–	–	25
*L. glabra*	138	18	–	–	–
*L. ovata*	159	7	–	–	–
*L. ventricosa*	85	36	–	–	–
*M. tuberculata*	59	–	6	–	–
*O. glabra*	461	24	–	–	–
*Ph. Acuta*	187	5	–		–
*Ps. columella*	1,108	58	–	–	–
*R. natalensis*	30	0	0	–	–
*T. granifera*	25	–	0	–	–

#### An overall experimental infection rate of amphistome species in freshwater snails

The pooled experimental infection rate of amphistome species among freshwater snails was 40% (95% CI: 18 to 65%) (Supplementary Figure S4). The results from the quality effects model revealed high heterogeneity (*Q* = 5370.79, *p* < 0.001), with *I*^2^ = 99% (Supplementary Figure S4).

#### Experimental infection rate of amphistomes in freshwater snail by continent

The pooled infection rate of amphistome species differed across continents (Figures 3A–C). Americas showed the highest pooled infection rate of 66% (95% CI: 26–96%, Figure 3B) of amphistome species among freshwater snails, followed by 21% in Europe (95% CI: 0–54%, Figure 3C) and 14% in Africa (95% CI: 0–38%, Figure 3A). A significant degree of heterogeneity was demonstrated; *I*^2^ was 99% for both Americas and Europe and 97% for Africa.

#### Experimental infection rate of amphistomes in freshwater snails by amphistome species

The pooled infection rate of individual amphistome species among intermediate host snail species from experimental infections was computed and presented in Table 6. The highest infection rate was caused by *P. cervi* with an overall infection rate of 65% (95% CI: 12–100%), followed by *Z. lunata* with 41% (95% CI: 11–75%), *C. microbothrium with* 22% (95% CI: 5–47%), and finally *C. daubneyi with* 11% (95% CI: 1–28%). The results from the quality effects model revealed a high degree of heterogeneity between studies on amphistome species (*I*^2^ > 95%, *p* < 0.01; Table 6).

**Table 6:** Pooled infection rate of amphistome species in snails from experimental infections across different continents (1984–2023).

Amphistome species	Continent	No of studies	No of snail hosts	PP (95% CI)	*I*^2^ (%)
*C. daubneyi*	Europe, Africa, South America	9	8	21 (3–45)	99
*C. microbothrium*	Africa	4	9	22 (5–47)	96
*P. cervi*	North America	1	3	68 (16–100)	100
*Z. lunata*	South America and North America	2	7	41 (11–75)	96

#### Experimental infection rate of amphistomes by snail species

Among the snails from the experimental infection, *G. truncatula* infected with *C*. *daubneyi and P. cervi* and *B. tropicus* infected with *C. microbothrium* qualified for the meta-analysis with an overall infection rate of 66% (95% CI: 34–92%) and 30% (95% CI: 0–74%), respectively. Furthermore, the results from the quality effects model revealed a high degree of heterogeneity between studies on snail infections (*I*^2^ > 99%, *p* < 0.01) for *G. truncatula* and *B. tropicus* (*I*^2^ > 98%, *p* < 0.01). Most infected snails were investigated in a few studies; hence, it was not possible to carry out a meta-analysis on them, and the frequency of infected snails with amphistomes is presented in Table 4.

#### Experimental infection rate of amphistomes in freshwater by time in years

Supplementary Figures S5A–D display the pooled experimental infection rate of amphistomes by period. The highest pooled infection rate was observed between 1984 and 1993 at 61% (95% CI: 15–100%) (Supplementary Figure S5A), followed by the years between 2004 and 2013 at 39% (95% CI: 4–82%) (Supplementary Figure S5C), 1994–2003 at 28% (95% CI: 10–51%) (Supplementary Figure S5B), and the lowest from 2014 to 2023 at 5% (95% CI: 2–9%) (Supplementary Figure S5D). The results from the quality effects model revealed a high heterogeneity between studies at each given time period, between 1984 and 1993 (*I*^2^ = 100%), between 1994 and 2003 (*I*^2^ = 98%), between 2004 and 2013 (*I*^2^ = 99%), and between 2014 and 2023 (*I*^2^ = 69%), (Supplementary Figures S5A–D).

#### Experimental infection rate of amphistomes in freshwater snails by detection techniques

**Figure 5:**
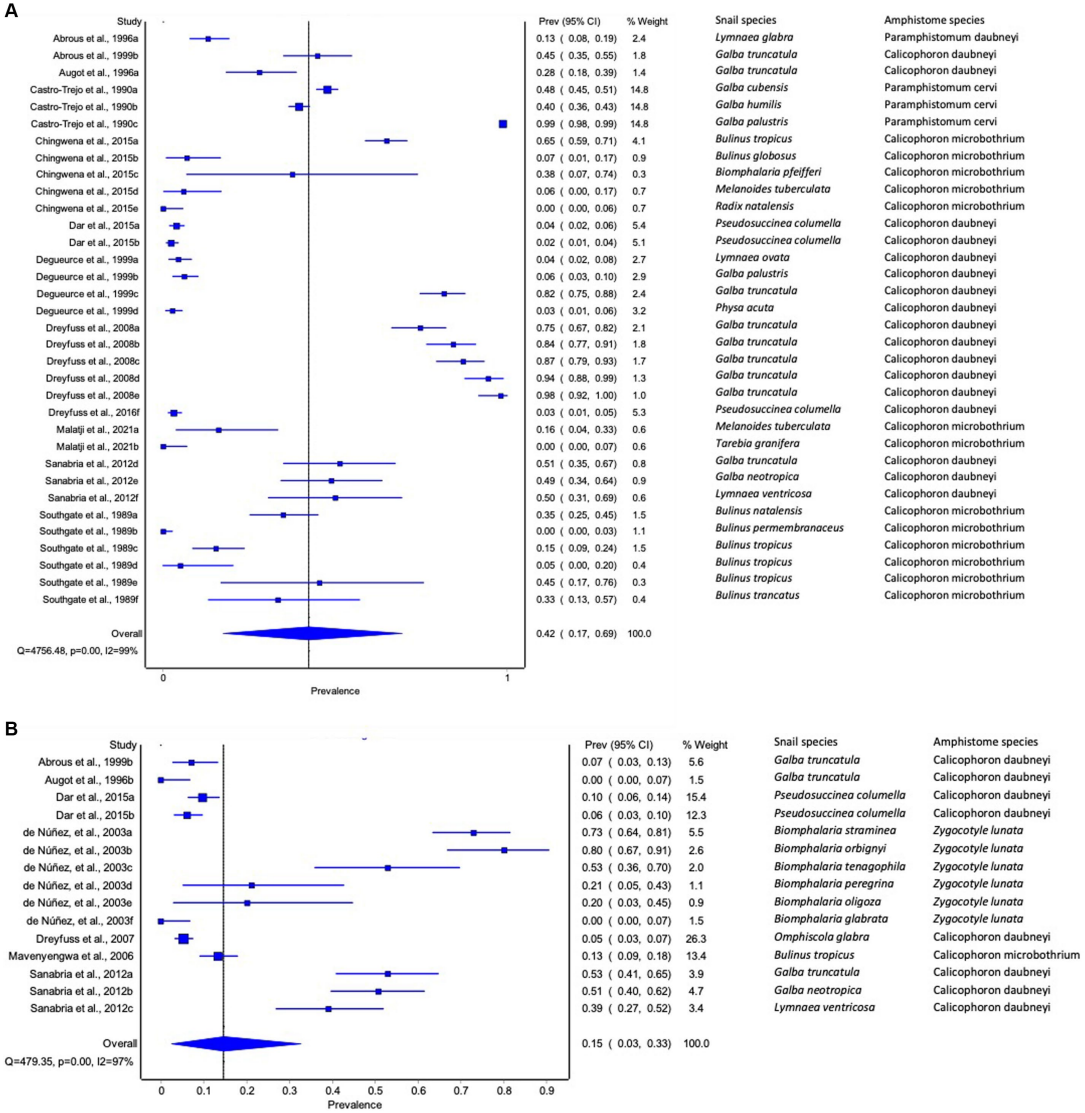
Forest plots of experimental infection rate of amphistome species in intermediate host snails as determined by **(A)** dissection and **(B)** shedding.

The estimated pooled infection rate of amphistome species obtained by shedding of cercariae was 15% (95% CI: 3–33%) in contrast to 42% (95% CI: 17–69%) obtained by dissection techniques (Figures 5A,B). The results from the quality effects model revealed a high heterogeneity between shedding (*I*^2^ = 97%) and dissection (*I*^2^ = 99%) (Figures 5A,B).

#### Publication bias of studies reporting on the experimental infections of amphistomes in intermediate host snails

Publication bias analysis revealed asymmetric funnel plots (Supplementary Figure S6), which indicated a small sample size bias or publication bias in the articles.

## Discussion

The results showed that field-based studies utilized a high number of snail species for assessing the prevalence of amphistome species in IHs. However, the prevalence of amphistome infection estimated from experimental infection was higher than that from natural infection. The variation may be due to differences in the environmental conditions set up, where the experimental conditions might have favored the success of the infections (18, 26). Dar et al. (27) also suggested that the higher infection rate of amphistome in snails from experimental infection studies may have been attributed to the decrease in snail’s resistance to infection due to the experimental conditions of breeding snails in the laboratory.

The study also revealed significant differences in amphistome prevalence among different continents, albeit with a limited number of studies. Although considerable work has been done on taxonomic aspects of ruminant amphistomes in livestock and wildlife in Africa (28–32), limited to no focus has been given on the ecology of the parasites, particularly with reference to the prevalence rate in IHs despite the fact that diversity of amphistomes species and their IHs seem to be higher in the African continent than other continents.

The prevalence of specific amphistome species in snails were mostly limited to one continent following the distribution of their specific intermediate host. The availability and abundance of susceptible definitive hosts on distinct continents, as proposed by Pfukenyi and Mukaratirwa (1) and Sibula et al. (6), may account for the distribution and prevalence of amphistome species in different snail species on different continents. The distribution of amphistome and their snail intermediate hosts could have been attributed to variations in environment and climate across these regions (5). Furthermore, the variations in noted prevalence among different amphistome species in the reviewed studies could be due to differences in the life cycle and transmission dynamics of these parasite species, as stated by González-Warleta et al. (33).

The high overall prevalence of *C. daubneyi* in Europe may have been attributed to a higher number of studies conducted than in any other continent. This could also be attributed to the high endemicity of this amphistome species in Europe, where a higher prevalence of *C. daubneyi* was recorded in definitive hosts (34). Furthermore, *C. daubneyi* natural infections were detected in a number of snails, which included *G. truncatula* (35–46), *L. ovata* (38), *O. glabra* (35, 36, 46), and *Ph. acuta* (38) in Europe. Its ability to naturally infect a vast number of snail species was also noted in experimental infection, where it was implicated in nine snail species, making it the main rumen fluke of cattle, sheep, and goats in Europe (34, 47). The presence of this amphistome species and its diverse intermediate host snails in Europe may be linked to the introduction of *C. daubneyi* to Western Europe during the movement of livestock (35) and climate change (milder winters and higher rainfall), favoring the completion of the parasite lifecycle (42).

The highest prevalence of amphistome species in snails from North America was *S. subtriquetrus,* with an overall prevalence of 6% (95% CI: 0–20%). The presence of different suitable IHs and definitive hosts in North America (48, 49) might have contributed to the presence of *S. subtriquetrus. S. subtriquetrus* was documented in three IHs including *F. fragilis, He. anceps,* and *Ps. columella.* These amphistome species have been reported as the dominant parasite in *Castor canadensis* in North America (49), and this might have contributed to the overall high prevalence in snails. A high prevalence of *S. subtriquetrus* has also been reported in Poland (50, 51) and the Czech Republic (52, 53) in beavers, but no study reported the prevalence of IHs in the same countries.

The highest overall prevalence estimated in snails from Asia was for *P. epiclitum*, followed by *E. explanatum*. The known snail host that serves as IHs for *P. epiclitum* is *I. exustus,* which is primarily an Asiatic species, whose distribution includes India, Thailand, the Malay Peninsula, and Sumatra (54). It was not possible to conduct a meta-analysis for various amphistome species as they were surveyed only in a limited number of studies and on one host species. This was also observed with other intermediate host snails, with only *G. truncatula* infected with *C. daubneyi* qualifying for meta-analysis from natural infection and *G. truncatula* and *B. tropicus* from experimental infection, whereas other infected snails were investigated in various studies.

The results from the experimental infection show that some amphistome species are more infective to a wider range of IH species, while others are specific to certain species. Of these, *C. daubneyi* (14, 18, 27, 35, 38, 55–57), *C. microbothrium* (16, 58–60), *Z. lunata* (61, 62), and *P. cervi* (63) infected multiple (≥4) IHs. According to Richards and Shade (64), genetic variations within and between species may account for the variability observed in the degree of infection of each amphistome species to different snail host species.

The highest pooled prevalence of amphistome species in intermediate host snails was observed between 2004 and 2013 for natural infections and between 1984 and 1993 for experimental infections. However, the results also showed that the pooled prevalence of infection lowered with time, with both natural and experimental infections recording the lowest infection rate between 2014 and 2023. This may be due to greater awareness raised in snail control programs for natural infection. Arfaa (65) previously reported that snail control strategies, such as the Anti-Bilharziasis campaign, will subsequently result in a significant decrease in the rate of *Paramphistomum* infections, and this might be the case, as confirmed by our results. Furthermore, the consequences of climate change may potentially be related to the variation noted in the prevalence of amphistomes (5). The lowest pooled infection rate shown by the experimental infection could be explained by the observed scarcity of epidemiological studies that have been conducted and published in recent years.

There was a significant difference in the pooled prevalence of amphistome species results among detection techniques. Cercarial shedding resulted in low pooled prevalence from both natural and experimental infections in this meta-analysis. Although it is a common method used to detect infections due to its relative affordability and ease of use, several authors have criticized it as inaccurate as it underestimates the true prevalence in the case of prepatent infection in intermediate host snails (66–68). This was in line with Born-Torrijos et al. (67), who found that snail dissection yielded more accurate results and subsequently greater prevalence than those based on cercarial shedding. The author further demonstrated that PCR-based methods were more accurate in determining the infection levels (67), although the presence of DNA of an amphistome might not indicate the suitability of the snail as an intermediate host. This is consistent with the findings by Tigga et al. (68), who suggested that because PCR-based techniques are sensitive and economical for large-scale detection, molecular approaches will lead to actual or higher prevalence than the shedding and snail dissection. However, only a few reviewed studies used PCR-based techniques, and this may be due to the cost of equipment and reagents and the requirement of trained personnel to carry out molecular analysis (67).

A high level of heterogeneity and publication bias was observed across continents and among snails. This could be due to the differences in the seasons in which the data were collected, the study designs, and the lack of standard protocol in sample collection.

## Strengths and limitations

The primary strengths of this systematic review and meta-analysis were adherence to the principle of PRISMA guidelines, the application of inclusion and exclusion criteria, and the provision of up-to-date estimates based on a quality effects model. Heterogeneity was analyzed using subgroup analyses. In addition, to the best of our knowledge, this is the first study to document the pooled prevalence of amphistome species in snails from various continents in a broad research period (1984–2023).

Limitations noted from this review included publication bias. The heterogeneity of the included studies in terms of study design, detection method, and quantification methods made it difficult to achieve a consistent meta-analysis despite using a standardized analysis process. Several studies failed to provide complete details on the information on the prevalence of amphistome species among snail species, and hence, few studies were eligible worldwide. From the few studies reviewed, the number of published studies was not evenly distributed across continents. Meta-analysis could not be conducted on some snail species and amphistome species due to the limited number of studies carried out. Thus, in this review, the prevalence data may not fully represent the prevalence of amphistome species among freshwater snail species worldwide.

The majority of the studies included in this review used cercarial shedding rather than PCR for the detection of amphistome infection. Cercarial shedding is less sensitive to the detection of amphistomes due to its inherent limitation, which may result in a low prevalence of amphistomes among snail species across continents. The estimated pooled prevalence of amphistome infections among freshwater snails observed in this review might be an underestimate of the true infection prevalence, especially in Africa, where the diversity of both amphistome species and freshwater snails is known to be high.

## Conclusion

The review provides valuable insights into the prevalence of amphistome infections in snails across different IHs and continents. However, results indicated a paucity of information on the prevalence of amphistome species in IHs. Hence, future studies should focus on determining the geographical expansion and the prevalence of amphistome species and their intermediate snail hosts, accompanied by extensive experimental infection studies to determine the susceptibility of various freshwater snail species across different geographical regions.

## Data availability statement

The original contributions presented in the study are included in the article/Supplementary material, further inquiries can be directed to the corresponding author.

## Author contributions

IN: Writing – review & editing, Writing – original draft, Methodology, Investigation, Formal analysis, Data curation. MM: Writing – review & editing, Validation, Supervision, Conceptualization. SM: Writing – review & editing, Validation, Supervision, Conceptualization.

## Footnotes

^1^ http://www.epigear.com
