# Supplementary material for: The prevalence and infection rates of amphistome species in intermediate snail hosts: a systematic review and meta-analysis
Source: Front Vet Sci. 2024 Jun 17;11:1418979. doi: 10.3389/fvets.2024.1418979 (PMC11216035; doi:10.3389/fvets.2024.1418979)
Supplement: Supplementary file 1 [file Table_1.docx]

**SUPPLEMENTARY TABLE 1** Summary of eligible studies included in the meta-analysis on natural infections of snails with amphistome species from 1984-2023

| **References** | **Country** | **Snail species** | **Amphistome species** | **No examined** | **No infected** | **Prevalence (%)** | **Detection method** | **Quality score** |
| --- | --- | --- | --- | --- | --- | --- | --- | --- |
| Abrous et al. (26) | France | *Omphiscola glabra* | *Calicophoron daubneyi* | 2396 | 155 | 6.45 | Dissection | 5 |
| Abrous et al. (26) | France | *Galba truncatula* | *Calicophoron daubneyi* | 1778 | 288 | 16.2 | Dissection | 5 |
| Abrous et al. (27) | Europe | *Planorbis leucostoma* | *Calicophoron daubneyi* | 1841 | 0 | 0 | Dissection | 5 |
| Abrous et al. (27) | Europe | *Omphiscola glabra* | *Calicophoron daubneyi* | 1719 | 7 | 0.41 | Dissection | 5 |
| Abrous et al. (27) | France | *Galba truncatula* | *Calicophoron daubneyi* | 1424 | 24 | 1.69 | Dissection | 5 |
| Abrous et al. (26) | France | *Galba truncatula* | *Calicophoron daubneyi* | 50 | 0 | 0 | Dissection | 5 |
| Ates and Umur (28) | Turkey | *Galba truncatula* | *Calicophoron daubneyi* | 300 | 12 | 4 | Dissection | 5 |
| Ates and Umur (28) | Turkey | *Physella acuta* | *Calicophoron daubneyi* | 200 | 0 | 0 | Dissection | 5 |
| Ates and Umur (28) | Turkey | *Planorbis planorbis* | *Calicophoron daubneyi* | 50 | 0 | 0 | Dissection | 5 |
| Bauri et al. (29) | India | *Gyraulus convexiusculus* | *Explanatum explanatum* | 173 | 21 | 12.14 | Molecular | 5 |
| Bauri et al. (29) | India | *Gyraulus convexiusculus* | *Explanatum explanatum* | 1440 | 30 | 2.1 | Cercariae shedding | 5 |
| Bauri et al. (29) | India | *Indoplanorbis exustus* | *Paramphistomum epiclitum* | 100 | 10 | 10 | Molecular | 5 |
| Bauri et al. (29) | India | *Indoplanorbis exustus* | *Paramphistomum epiclitum* | 3249 | 225 | 6.93 | Cercariae shedding | 5 |
| Bauri et al. (29) | India | *Lymnaea luteola* | *Fischoederius elongatus* | 100 | 20 | 20 | Molecular | 5 |
| Bauri et al. (29) | India | *Lymnaea luteola* | *Fischoederius elongatus* | 2361 | 17 | 0.72 | Shedding | 5 |
| Ostrowski de Núñez et al. (30) | Argentina | *Biomphalaria peregrina* | *Zygocotyle lunata* | 1424 | 8 | 0.56 | Shedding | 5 |
| Ostrowski de Núñez et al. (30) | Argentina | *Biomphalaria tenagophila* | *Zygocotyle lunata* | 4448 | 10 | 0.22 | Cercariae shedding | 5 |
| Degueurce et al. (31) | France | *Lymnaea ovata* | *Calicophoron daubneyi* | 108 | 4.63 | 4.63 | Dissection | 5 |
| Degueurce et al. (31) | France | *Galba truncatula* | *Calicophoron daubneyi* | 503 | 77 | 15.31 | Dissection | 5 |
| D Degueurce et al. (31) | France | *Physa acuta* | *Calicophoron daubneyi* | 230 | 7 | 3.04 | Dissection | 5 |
| Dreyfuss et al. (32) | France | *Galba truncatula* | *Calicophoron daubneyi* | 30594 | 2784 | 9.1 | Dissection | 5 |
| Flowers (33) | USA | *Menetus dilatat*us | *Pisciamphistoma stunkarkdi* | 494 | 4 | 0.81 | Shedding | 5 |
| Flowers (33) | USA | *Helisoma anceps* | *Stichorchis subtriquetrus* | 233 | 1 | 0.43 | Cercariae shedding | 5 |
| Flowers (33) | USA | *Pseudosuccinea columella* | *Stichorchis subtriquetrus* | 971 | 1 | 0.10 | Cercariae shedding | 5 |
| Flowers (33) | USA | *Ferrissia fragilis* | *Stichorchis subtriquetrus* | 243 | 5 | 2.06 | Cercariae shedding | 5 |
| Flowers (33) | USA | *Menetus dilatatus* | *Pisciamphistoma stunkardi* | 494 | 4 | 0.81 | Cercariae shedding | 5 |
| [Iglesias-Piñeiro et al. (34)](https://pubmed.ncbi.nlm.nih.gov/?term=Iglesias-Pi%C3%B1eiro%20J%5BAuthor%5D) | Spain | *Galba truncatula* | *Calicophoron daubneyi* | 1141 | 93 | 8.15 | Dissection | 5 |
| Jones et al. (35) | UK | *Galba truncatula,* | *Calicophoron daubneyi* | 892 | 36 | 4 | Molecular | 5 |
| Jones et al., (36) | UK | *Galba truncatula* | *Calicophoron daubneyi* | 134 | 15 | 11.19 | Dissection | 5 |
| Krailas et al. (37) | Thailand | *Melanoides tuberculata* | *Gastrothylax crumenifer* | 32026 | 8 | 0.02 | Cercariae shedding | 5 |
| Mage et al. (38) | France | *Galba truncatula* | *Calicophoron daubneyi* | 654 | 0 | 0 | Dissection | 5 |
| Mage et al. (38) | France | *Galba truncatula* | *Calicophoron daubneyi* | 798 | 6 | 0.8 | Dissection | 5 |
| Mage et al. (38) | France | *Galba truncatula* | *Calicophoron daubneyi* | 668 | 11 | 1.6 | Dissection | 5 |
| Mage et al. (38) | France | *Galba truncatula* | *Calicophoron daubneyi* | 934 | 19 | 2.1 | Dissection | 5 |
| Mage et al. (38) | France | *Galba truncatula* | *Calicophoron daubneyi* | 2 072 | 89 | 4.3 | Dissection | 5 |
| Mage et al. (38) | France | *Galba truncatula* | *Calicophoron daubneyi* | 1 543 | 46 | 3 | Dissection | 5 |
| Mage et al. (38) | France | *Galba truncatula* | *Calicophoron daubneyi* | 1 347 | 47 | 3.5 | Dissection | 5 |
| Mage et al. (38) | France | *Galba truncatula* | *Calicophoron daubneyi* | 2 477 | 156 | 6.3 | Dissection | 5 |
| Mage et al. (38) | France | *Galba truncatula* | *Calicophoron daubneyi* | 1 843 | 77 | 4.2 | Dissection | 5 |
| Mage et al. (38) | France | *Galba truncatula* | *Calicophoron daubneyi* | 1 475 | 54 | 3.7 | Dissection | 5 |
| Mage et al. (38) | France | *Galba truncatula* | *Calicophoron daubneyi* | 2 198 | 105 | 4.8 | Dissection | 5 |
| Mage et al. (38) | France | *Galba truncatula* | *Calicophoron daubneyi* | 2 782 | 147 | 5.3 | Dissection | 5 |
| Martínez-Ibeas et al. (39) | Spain | *Galba truncatula* | *Calicophoron daubneyi* | 230 | 44 |  | Cercariae shedding | 5 |
| Mattison et al. (40) | India | *Bithynia tentaculata* | *Gastrothylax crumenifer* | 921 | 5 | 22.54 | Dissection | 5 |
| Mattison et al. (40) | India | *Gyraulus convexiusculus* | *Explanatum explanatum* | 2532 | 59 | 3.15 | Dissection | 5 |
| Mattison et al. (40) | India | *Gyraulus convexiusculus* | *Gastrothylax crumenifer* | 2532 | 59 | 2.33 | Dissection | 5 |
| Mattison et al. (40) | India | *Helicorbis coenosus* | *Paramphistomum epiclitum* | 474 | 0 | 0.54 | Dissection | 5 |
| Mattison et al. (40) | India | *Helicorbis coenosus* | *Explanatum explanatum* | 474 | 0 | 0.44 | Dissection | 5 |
| Mattison et al. (40) | India | *Helicorbis coenosus* | *Fischoederius elongatus* | 474 | 0 | 0 | Dissection | 5 |
| Mattison et al. (40) | India | *Helicorbis coenosus* | *Gastrothylax crumenifer* | 474 | 0 | 0 | Dissection | 5 |
| Mattison et al. (40) | India | *Indoplanorbis exustus* | *Paramphistomum epiclitum* | 883 | 199 | 22.54 | Dissection | 5 |
| Mattison et al. (40) | India | *Lymnaea acuminata* | *Paramphistomum epiclitum* | 699 | 0 | 0 | Dissection | 5 |
| Mattison et al. (40) | India | *Lymnaea acuminata* | *Explanatum explanatum* | 699 | 0 | 0 | Dissection | 5 |
| Mattison et al. (40) | India | *Lymnaea acuminata* | *Fischoederius elongatus* | 699 | 0 | 0 | Dissection | 5 |
| Mattison et al. (40) | India | *Lymnaea acuminata* | *Gastrothylax crumenifer* | 699 | 0 | 0 | Dissection | 5 |
| Mattison et al. (40) | India | *Lymnaea luteola* | *Fischoederius elongatus* | 603 | 19 | 3.15 | Dissection | 5 |
| Mattison et al. (40) | India | *Vivipara bengalensis* | *Paramphistomum epiclitum* | 279 | 0 | 0 | Dissection | 5 |
| Mattison et al. (40) | India | *Vivipara bengalensis* | *Explanatum explanatum* | 279 | 0 | 0 | Dissection | 5 |
| Mattison et al. (40) | India | *Vivipara bengalensis* | *Fischoederius elongatus* | 279 | 0 | 0 | Dissection | 5 |
| Mattison et al. (40) | India | *Vivipara bengalensis* | *Gastrothylax crumenifer* | 279 | 0 | 0 | Dissection | 5 |
| Mattison et al. (40) | India | *Bithynia tentaculata* | *Orthocoelium scoliocoelium* | 2259 | 10 | 0.44 | Dissection | 5 |
| Mattison et al. (40) | India | *Helicorbis coenosus* | *Orthocoelium scoliocoelium* | 474 | 0 | 0 | Dissection | 5 |
| Mattison et al. (40) | India | *Lymnaea acuminata* | *Orthocoelium scoliocoelium* | 699 | 0 | 0 | Dissection | 5 |
| Mattison et al. (40) | India | *Vivipara bengalensis* | *Orthocoelium scoliocoelium* | 279 | 0 | 0 | Dissection | 5 |
| Rondelaud et al. (41) | France | *Galba truncatula* | *Calicophoron daubneyi* | 11025 | 126 | 1.14 | Dissection | 5 |
| Rondelaud et al. (42) | France | *Galba truncatula* | *Calicophoron daubneyi* | 3893 | 0 | 0 | Dissection | 5 |
| Rondelaud et al. (42) | France | *Galba truncatula* | *Calicophoron daubneyi* | 3897 | 131 | 3.36 | Dissection | 5 |
| Rondelaud et al. (42) | France | *Galba truncatula* | *Calicophoron daubneyi* | 1950 | 142 | 7.28 | Dissection | 5 |
| Rondelaud et al. (42) | France | *Omphiscola glabra* | *Calicophoron daubneyi* | 3900 | 0 | 0 | Dissection | 5 |
| Rondelaud et al. (42) | France | *Omphiscola glabra* | *Calicophoron daubneyi* | 3900 | 66 | 1.69 | Dissection | 5 |
| Rondelaud et al. (42) | France | *Omphiscola glabra* | *Calicophoron daubneyi* | 1948 | 42 | 2.16 | Dissection | 5 |
| Rondelaud et al. (43) | France | Galba truncatula | *Calicophoron daubneyi* | 19237 | 111 | 0.58 | Dissection | 5 |
| Southgate et al. (44) | Kenya | *Bulinus tropicus* | *Calicophoron microbothrium* | 55 | 9 | 16.36 | Dissection | 5 |
| Southgate et al. (45) | Kenya | *Bulinus tropicus* | *Calicophoron microbothrium* | 351 | 75 | 21.37 | Cercariae shedding | 5 |

USA – United States of America; UK – United Kingdom

**SUPPLEMENTARY TABLE 2** Summary of eligible studies included in the meta-analysis on experimental infection of snails with amphistome species from 1984-2023

| **References** | **Country** | **Snail species** | **Amphistome species** | **No examined** | **No infected** | **Prevalence (%)** | **Detection method** | **Quality score** |
| --- | --- | --- | --- | --- | --- | --- | --- | --- |
| Abrous et al. (46) | France | *Omphiscola glabra* | *Calicophoron daubneyi* | 138 | 18 | 13.04 | Dissection | 5 |
| Abrous et al. (26) | France | *Galba truncatula* | *Calicophoron daubneyi* | 98 | 44 | 44.9 | Dissection | 5 |
| Abrous et al. (26) | France | *Galba truncatula* | *Calicophoron daubneyi* | 98 | 7 | 7.14 | Cercariae shedding | 5 |
| Augot et al. (47) | France | *Galba truncatula* | *Calicophoron daubneyi* | 75 | 21 | 28 | Dissection | 5 |
| Augot et al. (47) | France | *Galba truncatula* | *Calicophoron daubneyi* | 25 | 0 | 0 | Cercariae shedding | 5 |
| Castro-Trejo et al. (48) | Mexico | *Galba cubensis* | *Paramphistomum cervi* | 1000 | 477 | 47.7 | Dissection | 5 |
| Castro-Trejo et al. (48) | Mexico | *Galba humilis* | *Paramphistomum cervi* | 1000 | 395 | 39.5 | Dissection | 5 |
| Castro-Trejo et al. (48) | Mexico | *Galba palustris* | *Paramphistomum cervi* | 1000 | 987 | 98.7 | Dissection | 5 |
| Chingwena et al. (16) | Zimbabwe | *Bulinus tropicus* | *Calicophoron microbothrium* | 240 | 156 | 65 | Dissection | 5 |
| Chingwena et al. (16) | Zimbabwe | *Bulinus globosus* | *Calicophoron microbothrium* | 44 | 3 | 6.82 | Dissection | 5 |
| Chingwena et al. (16) | Zimbabwe | *Biomphalaria pfeifferi* | *Calicophoron microbothrium* | 8 | 3 | 37.5 | Dissection | 5 |
| Chingwena et al. (16) | Zimbabwe | *Melanoides tuberculata* | *Calicophoron microbothrium* | 34 | 2 | 5.88 | Dissection | 5 |
| Chingwena et al. (16) | Zimbabwe | *Radix natalensis* | *Calicophoron microbothrium* | 30 | 0 | 0 | Dissection | 5 |
| Dar et al. (49) | Egypt | *Pseudosuccinea columella* | *Calicophoron daubneyi* | 320 | 12 | 3.75 | Dissection | 5 |
| Dar et al. (49) | Egypt | *Pseudosuccinea columella* | *Calicophoron daubneyi* | 269 | 26 | 9.67 | Cercariae shedding | 5 |
| Dar et al. (49) | France | *Pseudosuccinea columella* | *Calicophoron daubneyi* | 304 | 7 | 2.3 | Dissection | 5 |
| Dar et al. (49) | France | *Pseudosuccinea columella* | *Calicophoron daubneyi* | 215 | 13 | 6.05 | Cercariae shedding | 5 |
| Ostrowski de Núñez et al. (50) | Argentina | *Biomphalaria straminea* | *Zygocotyle lunata* | 96 | 70 | 72.92 | Cercariae shedding | 5 |
| Ostrowski de Núñez et al. (50) | Argentina | *Biomphalaria orbignyi* | *Zygocotyle lunata* | 45 | 36 | 80 | Cercariae shedding | 5 |
| Ostrowski de Núñez et al. (50) | Argentina | *Biomphalaria tenagophila* | *Zygocotyle lunata* | 34 | 18 | 52.94 | Cercariae shedding | 5 |
| Ostrowski de Núñez et al. (50) | Argentina | *Biomphalaria peregrina* | *Zygocotyle lunata* | 19 | 4 | 21.05 | Cercariae shedding | 5 |
| Ostrowski de Núñez et al. (50) | Argentina | *Biomphalaria oligoza* | *Zygocotyle lunata* | 15 | 3 | 20 | Cercariae shedding | 5 |
| Ostrowski de Núñez et al. (50) | Argentina | *Biomphalaria glabrata* | *Zygocotyle lunata* | 25 | 0 | 0 | Cercariae shedding | 5 |
| Ostrowski de Núñez et al. (50) | France | *Lymnaea ovata* | *Calicophoron daubneyi* | 159 | 7 | 4.4 | Dissection | 5 |
| Degueurce et al. (31) | France | *Galba palustris* | *Calicophoron daubneyi* | 166 | 10 | 6.02 | Dissection | 5 |
| Degueurce et al. (31) | France | *Galba truncatula* | *Calicophoron daubneyi* | 136 | 111 | 81.62 | Dissection | 5 |
| Degueurce et al. (31) | France | *Physa acuta* | *Calicophoron daubneyi* | 187 | 5 | 2.67 | Dissection | 5 |
| Dreyfuss et al. (51) | Czech Republic | *Omphiscola glabra* | *Calicophoron daubneyi* | 461 | 24 | 5.21 | Cercariae shedding | 5 |
| Dreyfuss et al. (32) | France | *Galba truncatula* | *Calicophoron daubneyi* | 119 | 89 | 74.79 | Dissection | 5 |
| Dreyfuss et al. (32) | France | *Galba truncatula* | *Calicophoron daubneyi* | 102 | 86 | 84.31 | Dissection | 5 |
| Dreyfuss et al. (32) | France | *Galba truncatula* | *Calicophoron daubneyi* | 93 | 81 | 87.1 | Dissection | 5 |
| Dreyfuss et al. (32) | France | *Galba truncatula* | *Calicophoron daubneyi* | 72 | 68 | 94.44 | Dissection | 5 |
| Dreyfuss et al. (32) | France | *Galba truncatula* | *Calicophoron daubneyi* | 51 | 50 | 98.04 | Dissection | 5 |
| Dreyfuss et al. (52) | France | *Pseudosuccinea columella* | *Calicophoron daubneyi* | 319 | 10 | 3.13 | Dissection | 5 |
| Etges (53) | USA | *Helisoma anceps* | *Zygocotyle lunata* | 127 | 25 | 19.69 | Dissection and cercariae shedding | 5 |
| Malatji et al. (54) | South Africa | *Melanoides tuberculata* | *Calicophoron microbothrium* | 25 | 4 | 16 | Dissection | 5 |
| Malatji et al. (54) | South Africa | *Tarebia granifera* | *Calicophoron microbothrium* | 25 | 0 | 0 | Dissection | 5 |
| Mavenyengwa et al (55) | Zimbabwe | *Bulinus tropicus* | *Calicophoron microbothrium* | 235 | 31 | 13.19 | Cercariae shedding | 5 |
| Rondelaud et al. (56) | France | *Galba truncatula* | *Paramphistomum daubneyi* | 83 | 59 | 71.08 | Dissection and cercariae shedding | 5 |
| Sanabria et al. (18) | Argentina | *Galba truncatula* | *Calicophoron daubneyi* | 68 | 36 | 52.94 | Cercariae shedding | 5 |
| Sanabria et al. (18) | Argentina | *Galba neotropica* | *Calicophoron daubneyi* | 81 | 41 | 50.62 | Cercariae shedding | 5 |
| Sanabria et al. (18) | Argentina | *Lymnaea ventricosa* | *Calicophoron daubneyi* | 59 | 23 | 38.98 | Cercariae shedding | 5 |
| Sanabria et al. (18) | Argentina | *Galba truncatula* | *Calicophoron daubneyi* | 37 | 19 | 51.35 | Dissection | 5 |
| Sanabria et al. (18) | Argentina | *Galba neotropica* | *Calicophoron daubneyi* | 45 | 22 | 48.89 | Dissection | 5 |
| Sanabria et al. (18) | Argentina | *Lymnaea ventricosa* | *Calicophoron daubneyi* | 26 | 13 | 50 | Dissection | 5 |
| Southgate et al. (45) | South Africa | *Bulinus natalensis* | *Calicophoron microbothrium* | 89 | 31 | 34.83 | Dissection | 5 |
| Southgate et al. (45) | Kenya | *Bulinus permembranaceus* | *Calicophoron microbothrium* | 64 | 0 | 0 | Dissection | 5 |
| Southgate et al. (45) | Kenya | *Bulinus tropicus* | *Calicophoron microbothrium* | 91 | 14 | 15.38 | Dissection | 5 |
| Southgate et al. (45) | Zimbabwe | *Bulinus tropicus* | *Calicophoron microbothrium* | 20 | 1 | 5 | Dissection | 5 |
| Southgate et al. (45) | South Africa | *Bulinus tropicus* | *Calicophoron microbothrium* | 11 | 5 | 45.45 | Dissection | 5 |
| Southgate et al. (45) | Gabon | *Bulinus trancatus* | *Calicophoron microbothrium* | 18 | 6 | 33.33 | Dissection | 5 |
