# Supplementary material for: The prevalence and infection rates of amphistome species in intermediate snail hosts: a systematic review and meta-analysis
Source: Front Vet Sci. 2024 Jun 17;11:1418979. doi: 10.3389/fvets.2024.1418979 (PMC11216035; doi:10.3389/fvets.2024.1418979)
Supplement: Supplementary file 2 [file Image_1.pdf]

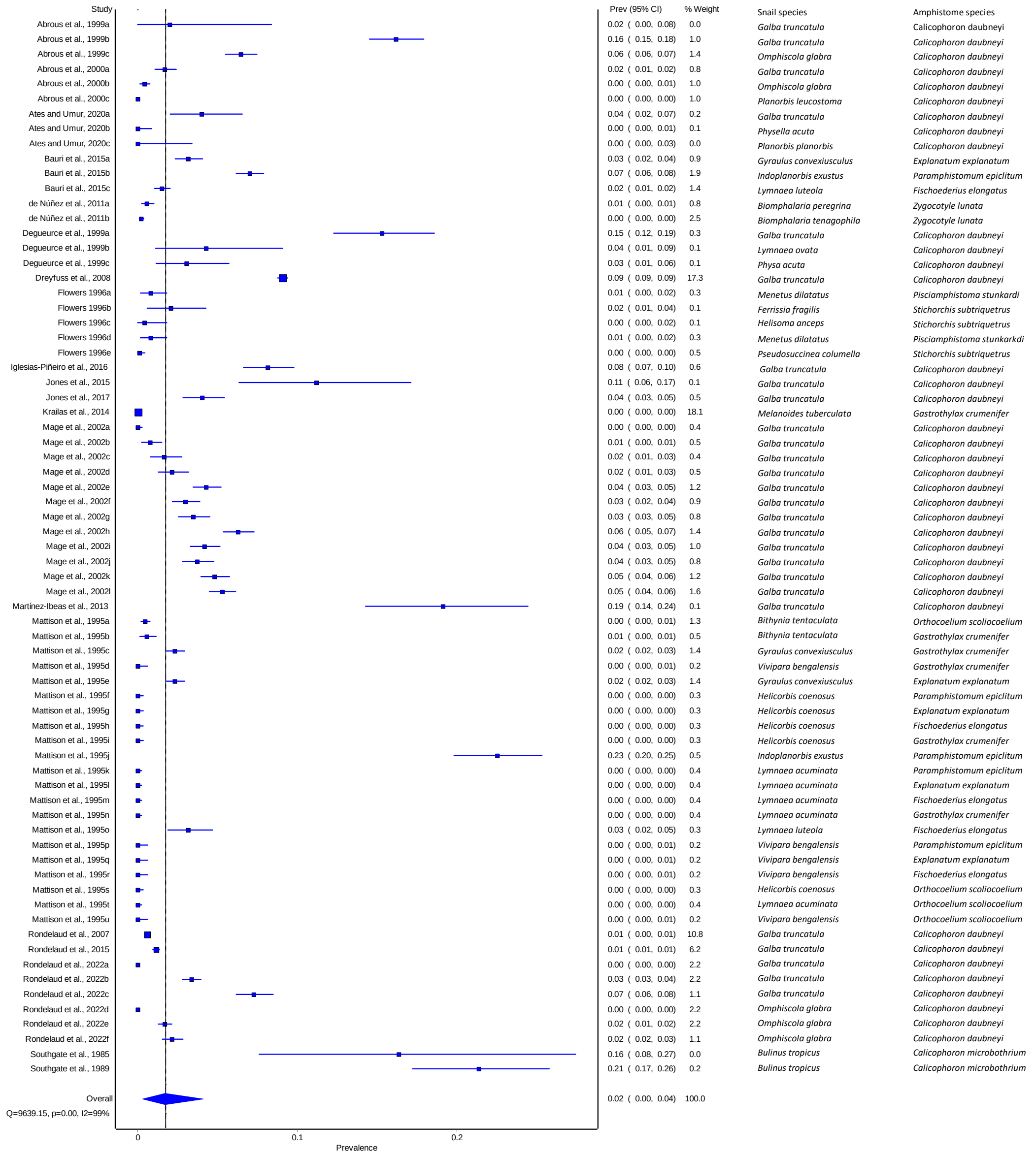

SUPPLEMENTARY FIGURE 1

Forest plots of the overall prevalence of natural infections of amphistome species in snail intermediate hosts species recorded from 1984 to 2023.

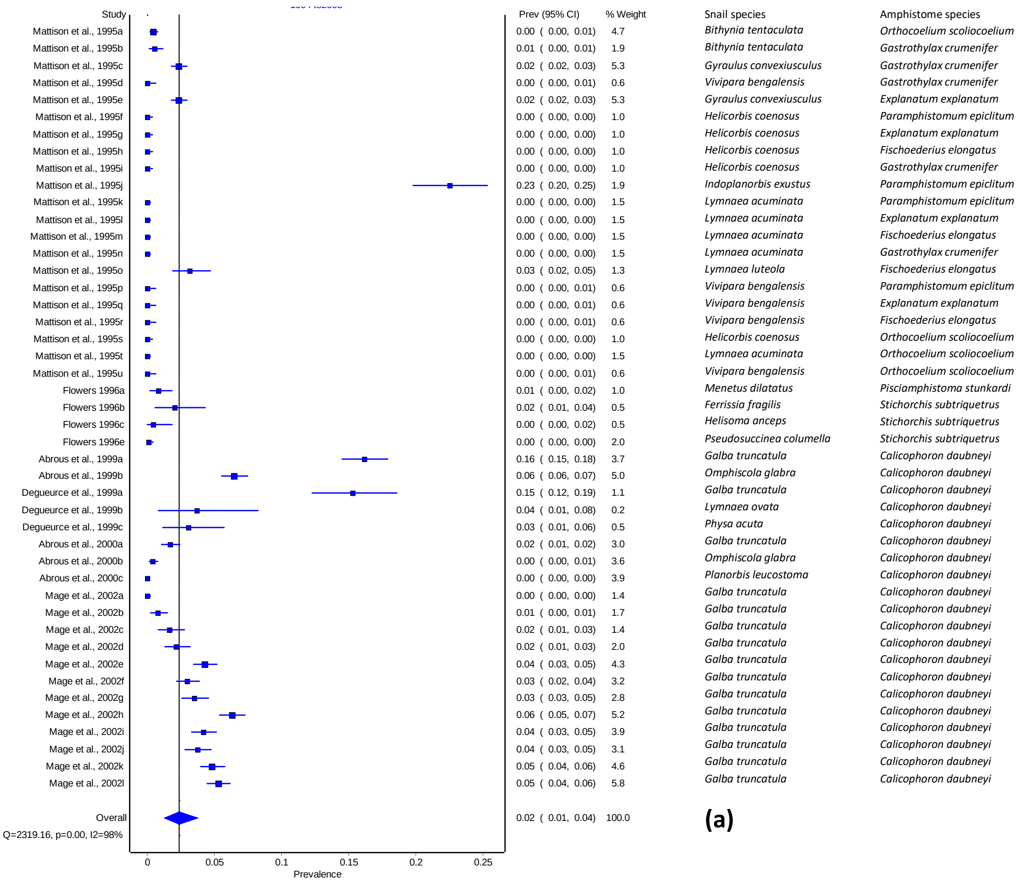

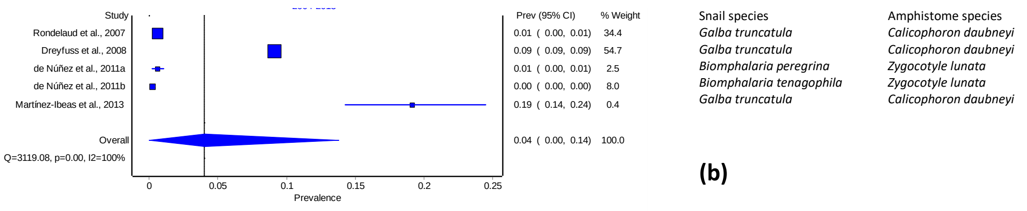

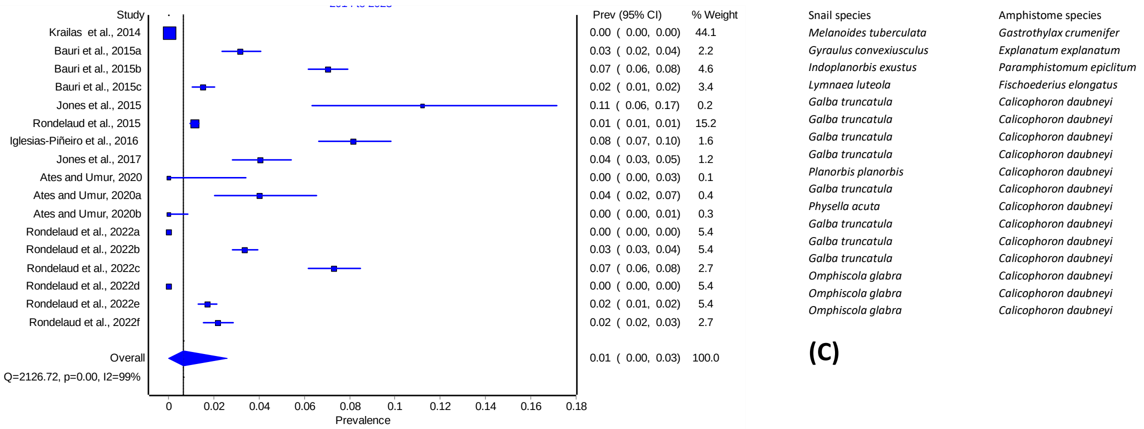

SUPPLEMENTARY FIGURE 2

Forest plots of prevalence of natural infections of amphistome species in snail intermediate hosts (a) between 1994 and 2003, (b) between 2004 and 2013 and (c) between 2014 and 2023

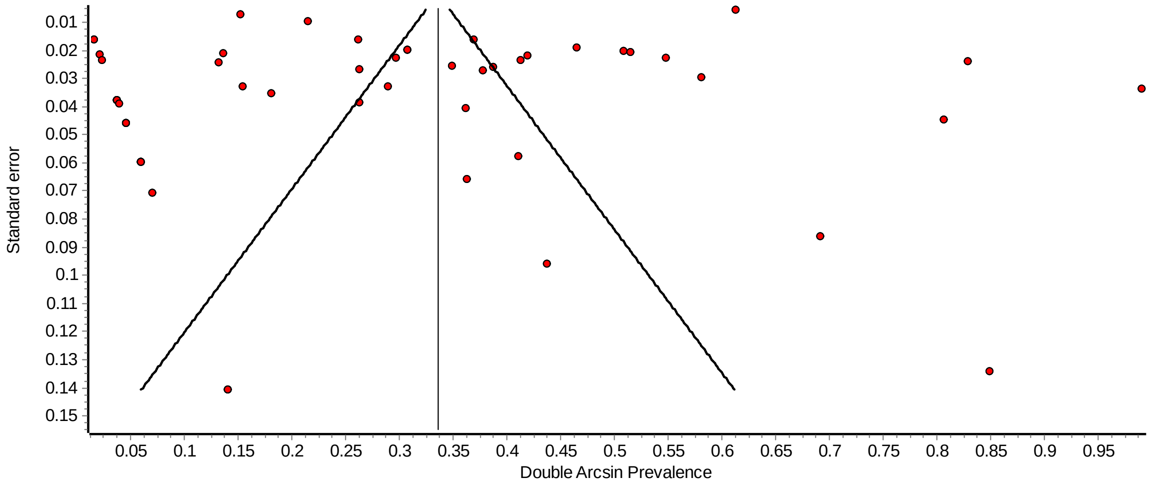

SUPPLEMENTARY FIGURE 3

Funnel plot with 95% confidence limit showing publication bias across studies on the prevalence of amphistome species among snail host species in natural infections.

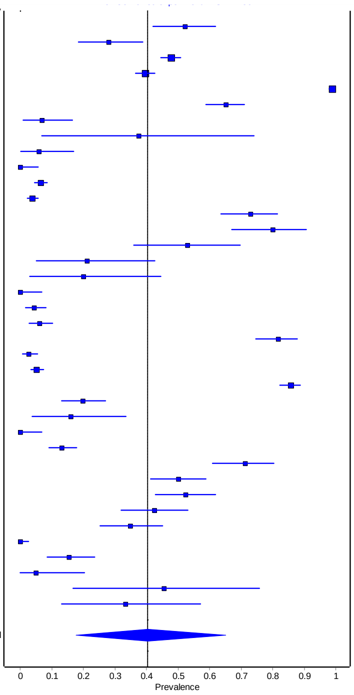

Study	Prev (95% CI)	% Weight	Snail species	Amphistome species
Abrous et al., 1999	0.52 (0.42, 0.62)	1.3	*Lymnaea glabra*	*Calicophoron daubneyi*
Augot et al., 1996	0.28 (0.18, 0.39)	1.0	*Galba truncatula*	*Calicophoron daubneyi*
Castro-Trejo et al., 1990a	0.48 (0.45, 0.51)	13.3	*Galba truncatula*	*Calicophoron daubneyi*
Castro-Trejo et al., 1990b	0.40 (0.36, 0.43)	13.3	*Galba cubensis*	*Paramphistomum cervi*
Castro-Trejo et al., 1990c	0.99 (0.98, 0.99)	13.3	*Galba humilis*	*Paramphistomum cervi*
Chingwena et al., 2015a	0.65 (0.59, 0.71)	3.2	*Galba palustris*	*Paramphistomum cervi*
Chingwena et al., 2015b	0.07 (0.01, 0.17)	0.6	*Bulinus tropicus*	*Calicophoron microbothrium*
Chingwena et al., 2015c	0.38 (0.07, 0.74)	0.1	*Bulinus globosus*	*Calicophoron microbothrium*
Chingwena et al., 2015d	0.06 (0.00, 0.17)	0.5	*Biomphalaria pfeifferi*	*Calicophoron microbothrium*
Chingwena et al., 2015e	0.00 (0.00, 0.06)	0.4	*Melanoides tuberculata*	*Calicophoron microbothrium*
Dar et al., 2015a	0.06 (0.05, 0.09)	7.8	*Radix natalensis*	*Calicophoron microbothrium*
Dar et al., 2015b	0.04 (0.02, 0.06)	6.9	*Pseudosuccinea columella*	*Calicophoron daubneyi*
de Núñez et al., 2003a	0.73 (0.64, 0.81)	1.3	*Pseudosuccinea columella*	*Calicophoron daubneyi*
de Núñez et al., 2003b	0.80 (0.67, 0.91)	0.6	*Biomphalaria straminea*	*Zygocotyle lunata*
de Núñez et al., 2003c	0.53 (0.36, 0.70)	0.5	*Biomphalaria orbignyi*	*Zygocotyle lunata*
de Núñez et al., 2003d	0.21 (0.05, 0.43)	0.3	*Biomphalaria tenagophila*	*Zygocotyle lunata*
de Núñez et al., 2003e	0.20 (0.03, 0.45)	0.2	*Biomphalaria peregrina*	*Zygocotyle lunata*
de Núñez et al., 2003f	0.00 (0.00, 0.07)	0.3	*Biomphalaria oligoza*	*Zygocotyle lunata*
Degueurce et al., 1999a	0.04 (0.02, 0.08)	2.1	*Biomphalaria glabrata*	*Zygocotyle lunata*
Degueurce et al., 1999b	0.06 (0.03, 0.10)	2.2	*Lymnaea ovata*	*Calicophoron daubneyi*
Degueurce et al., 1999c	0.82 (0.75, 0.88)	1.8	*Galba palustris*	*Calicophoron daubneyi*
Degueurce et al., 1999d	0.03 (0.01, 0.06)	2.5	*Galba truncatula*	*Calicophoron daubneyi*
Dreyfuss et al., 2007	0.05 (0.03, 0.07)	6.1	*Physa acuta*	*Calicophoron daubneyi*
Dreyfuss et al., 2008	0.86 (0.82, 0.89)	5.8	*Omphiscola glabra*	*Calicophoron daubneyi*
Etges 1992	0.20 (0.13, 0.27)	1.7	*Galba truncatula*	*Calicophoron daubneyi*
Malatji et al., 2021a	0.16 (0.04, 0.33)	0.3	*Helisoma anceps*	*Zygocotyle lunata*
Malatji et al., 2021b	0.00 (0.00, 0.07)	0.3	*Melanoides tuberculata*	*Calicophoron microbothrium*
Mavenyengwa et al., 2006	0.13 (0.09, 0.18)	3.1	*Tarebia granifera*	*Calicophoron microbothrium*
Rondelaud et al., 2013	0.71 (0.61, 0.80)	1.1	*Bulinus tropicus*	*Calicophoron microbothrium*
Sanabria et al., 2012a	0.50 (0.41, 0.59)	1.7	*Galba truncatula*	*Calicophoron daubneyi*
Sanabria et al., 2012b	0.52 (0.43, 0.62)	1.4	*Galba neotropica*	*Calicophoron daubneyi*
Sanabria et al., 2012c	0.42 (0.32, 0.53)	1.1	*Galba truncatula*	*Calicophoron daubneyi*
Southgate et al., 1989a	0.35 (0.25, 0.45)	1.2	*Lymnaea ventricosa*	*Calicophoron daubneyi*
Southgate et al., 1989b	0.00 (0.00, 0.03)	0.9	*Bulinus natalensis*	*Calicophoron microbothrium*
Southgate et al., 1989c	0.15 (0.09, 0.24)	1.2	*Bulinus permembranaceus*	*Calicophoron microbothrium*
Southgate et al., 1989d	0.05 (0.00, 0.20)	0.3	*Bulinus tropicus*	*Calicophoron microbothrium*
Southgate et al., 1989e	0.45 (0.17, 0.76)	0.2	*Bulinus tropicus*	*Calicophoron microbothrium*
Southgate et al., 1989f	0.33 (0.13, 0.57)	0.2	*Bulinus tropicus*	*Calicophoron microbothrium*
			Bulinus trancatus	*Calicophoron microbothrium*
Overall	0.40 (0.18, 0.65)	100.0		

Q=5315.14, p=0.00, I2=99%

SUPPLEMENTARY FIGURE 4

Forest plots of the overall experimental infection rate of amphistome species in snail intermediate hosts species recorded from 1984 to 2023.

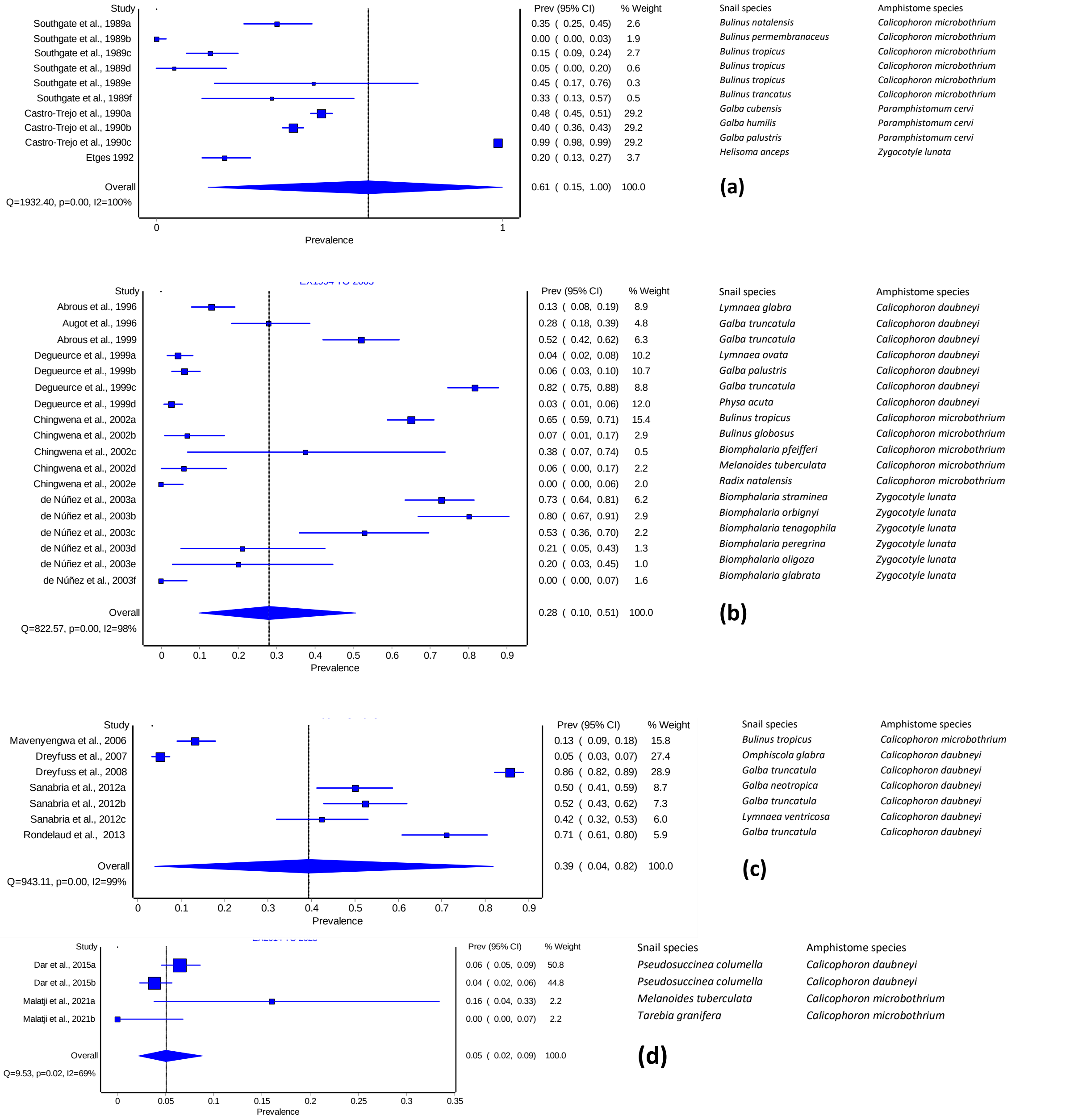

SUPPLEMENTARY FIGURE 5

Forest plots of experimental infection rate of amphistome species in snail intermediate hosts (a) between 1984 and 1993, (b) between 1994 and 2003 and (c) between 2004 and 2023

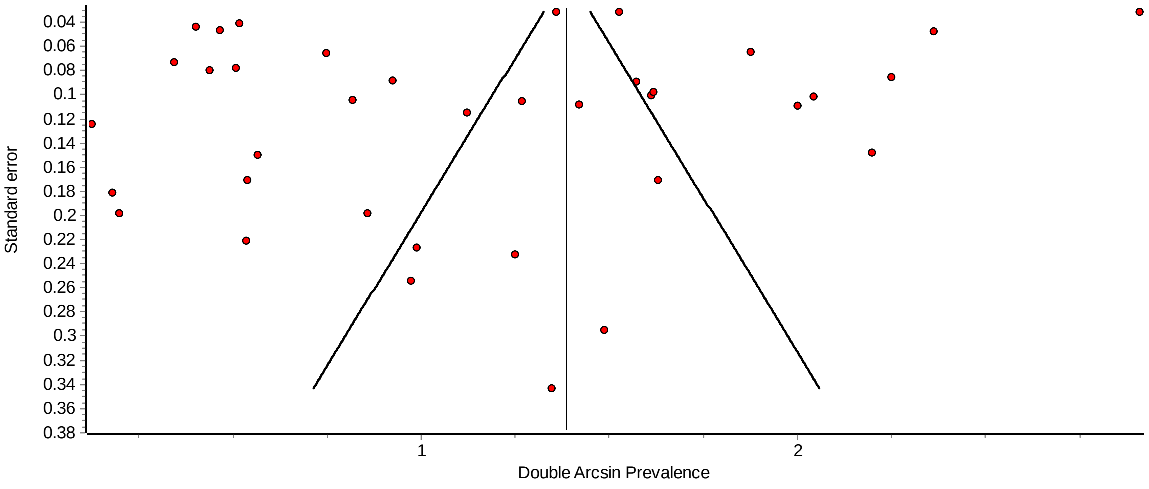

SUPPLEMENTARY FIGURE 6

Funnel plot with 95% confidence limit showing publication bias across studies on the infection rate of amphistome species among snail host species in experimental infections.
